# Silencing of ferrochelatase enhances 5-aminolevulinic acid-based fluorescence and photodynamic therapy efficacy

**DOI:** 10.1038/bjc.2011.12

**Published:** 2011-02-08

**Authors:** L Teng, M Nakada, S-G Zhao, Y Endo, N Furuyama, E Nambu, I V Pyko, Y Hayashi, J-I Hamada

**Affiliations:** 1 Department of Neurosurgery, Graduate School of Medical Science, Kanazawa University, 13-1 Takara-machi, Kanazawa, Ishikawa 920-8641, Japan; 2 Department of Neurosurgery, The First Clinical College of Harbin Medical University, Nangang Harbin 150001, People's Republic of China; 3 Central Research Resource Branch, Cancer Research Institute, Kanazawa University, Kanazawa, Ishikawa 920-1192, Japan

**Keywords:** glioma, ferrochelatase, 5-aminolevulinic acid, fluorescence, photodynamic therapy

## Abstract

**Background:**

Recurrence of glioma frequently occurs within the marginal area of the surgical cavity due to invading residual cells. 5-Aminolevulinic acid (5-ALA) fluorescence-guided resection has been used as effective therapeutic modalities to improve discrimination of brain tumour margins and patient prognosis. However, the marginal areas of glioma usually show vague fluorescence, which makes tumour identification difficult, and the applicability of 5-ALA-based photodynamic therapy (PDT) is hampered by insufficient therapeutic efficacy in glioma tissues.

**Methods:**

To overcome these issues, we assessed the expression of ferrochelatase (*FECH*) gene, which encodes a key enzyme that catalyses the conversion of protoporphyrin IX (PpIX) to heme, in glioma surgical specimens and manipulated FECH in human glioma cell lines.

**Results:**

Prominent downregulation of FECH mRNA expression was found in glioblastoma tissues compared with normal brain tissues, suggesting that FECH is responsible for PpIX accumulation in glioblastoma cells. Depletion of FECH by small interference RNA enhanced PpIX fluorescence after exposure to 5-ALA concomitant with increased intracellular PpIX accumulation in glioma cells. Silencing of FECH caused marked growth inhibition and apoptosis induction by PDT in glioma cells.

**Conclusion:**

These results suggest that knockdown of FECH is a potential approach to enhance PpIX fluorescent quality for optimising the subjective discrimination of vague fluorescence and improving the effect of 5-ALA-PDT.

Glioma is the most common primary malignant brain tumour in adults, characterised by rapid proliferation, marked infiltration, and poor prognosis (Lefranc *et al*, 2006; Iacob and Dinca, 2009). Recurrence of glioma occurs within ∼2 cm of the margins of the resected cavity due to its invasive character (Wallner *et al*, 1989; Aydin *et al*, 2001). Therefore, methods that easily detect tumour margins during the operation might be of benefit. 5-Aminolevulinic acid (5-ALA) fluorescence-guided resection of glioma is a rapidly growing novel approach to improve the extent of tumour resection with broad application in both pre-clinical and clinical settings (Stummer *et al*, 1998b, 1998c, 2000). Recent controlled phase III clinical trials demonstrated that this surgical method enables more complete resection of contrast-enhancing lesions compared with conventional microsurgery and improves progression-free survival in patients with malignant glioma (Stummer *et al*, 2006; Pichlmeier *et al*, 2008).

5-Aminolevulinic acid is not itself fluorescent, the metabolic precursor of heme in the heme biosynthesis pathway, which is metabolised into endogenous fluorescent protoporphyrin IX (PpIX) (Kennedy and Pottier, 1992; Loh *et al*, 1993; Peng *et al*, 1997; Gossner *et al*, 1998; Inoue *et al*, 2007). An oral-intake of 5-ALA resulted in accumulation of PpIX in malignant gliomas. Heme biosynthesis consists of a series of enzyme-catalyzed steps, the last of which is the conversion of PpIX to heme by ferrochelatase (FECH), which is located in the inner mitochondrial membrane (Ferreira *et al*, 1995; Kemmner *et al*, 2008). Exposure to an appropriate light source with the specific wavelength causes excitation of PpIX and allows it to become visible as red fluorescence with bimodal function; fluorescence marker, and 5-ALA mediated-photodynamic therapy (PDT). Red fluorescence exerts an effect as a discriminating marker to assist neurosurgeons to visualise the extent and margins of tumours (Stummer *et al*, 1998c, 2006). Photodynamic therapy results in a series of photochemical and photobiological events that cause irreversible damage to tumour cells. Nowadays, PDT is widely used as an effective adjuvant therapy for treating various cancers, including malignant glioma (Muller and Wilson, 1995; Kostron *et al*, 1996; Onuki *et al*, 2004).

However, successful glioma resection often depends on the neurosurgeon's ability to distinguish residual tumour tissue from surrounding brain tissue even under the assistant with PpIX fluorescence. The marginal area containing infiltrating glioma cells shows vague fluorescence because the density of the glioma cells is low and heterogeneous, resulting in insufficient 5-ALA uptake and PpIX accumulation (Utsuki *et al*, 2006). On the other hand, unfortunately, current 5-ALA–PDT protocols have yet to be widely established in clinical treatment for glioma. This may partly be due to limitations in current PDT regimens and partly due to the therapeutic efficacy of 5-ALA–PDT in pre-clinical settings (Norum *et al*, 2009). If the specificity and selectivity of 5-ALA–PDT could be improved in glioma tissue, 5-ALA–PDT would probably be regarded as a promising and competitive alternative in glioma treatment (Angell-Petersen *et al*, 2007). To improve the accuracy of vague fluorescence discrimination and 5-ALA–PDT efficacy, we attempted to accumulate intracellular PpIX under fixed 5-ALA concentrations and time intervals by manipulating *FECH* gene expression.

In this study, we examined FECH expression and localisation in glioma surgical specimens and the correlation between FECH expression and PpIX accumulation in glioma cell lines. To enhance 5-ALA fluorescence quality and 5-ALA–PDT sensitivity, we applied small interfering RNA (siRNA) of FECH to induce PpIX accumulation. The results suggest that FECH knockdown enhanced the molecular imaging quality of 5-ALA and the effect of 5-ALA–PDT.

## Materials and methods

### Antibodies and reagents

Cell culture media, sodium dodecyl sulphate (SDS)–polyacrylamide gel, polyvinylidene difluoride (PVDF) membrane, and bovine serum albumin (BSA) were purchased from Invitrogen (Carlsbad, CA, USA). Rabbit polyclonal antibody and mouse polyclonal antibody against FECH were purchased from LifeSpan Biosciences (Seattle, WA, USA) and Abcam (Cambridge, MA, USA), respectively. Small interfering RNA specific to human FECH and luciferase as a control was purchased from Qiagen (Gaithersburg, MD, USA). 5-Aminolevulinic acid hydrochloride was purchased from Sigma (St Louis, MO, USA) and dissolved in deionised water to make a 60-mM stock, maintained at −20°C away from light until use as described previously (Au *et al*, 2006).

### Clinical samples and histology

Following an institutional review board-approved protocol, fresh human brain tumour tissues were obtained from 66 patients who had been treated neurosurgically. Non-neoplastic control brain tissues were identified far from the tumour margins. Brain tumours were extirpated from the centre of the tumour, classified and characterised according to the revised World Health Organisation classification (Louis *et al*, 2007). The 66 gliomas consisted of 17 diffuse astrocytomas, 8 anaplastic astrocytomas, and 41 glioblastomas. All of the tumour tissues were obtained during the primary resection, and none of the patients had been subjected to chemotherapy or radiation therapy before the therapeutic removal of gliomas. Tissue samples were obtained and immediately frozen in liquid nitrogen. Samples were maintained at −80°C until use.

### Cell line and cell culture

Human glioma cell lines SNB19, U87 (American Type Culture Collection, Manassas, VA, USA), and G112 (Westphal *et al*, 1988; Giese *et al*, 1994) were cultured at 37°C in a humidified CO_2_ incubator with DMEM, 10% fetal bovine serum (FBS), and 1% penicillin–streptomycin. The cells were passaged twice weekly, and once they were nearly confluent, they were released with 0.25% trypsin–EDTA.

### Immunohistochemistry

Immunohistochemistry was done using an avidin–biotin immunoperoxidase technique to stain 5-*μ*m sections as previously described (Nakada *et al*, 2005). Rabbit polyclonal antibody against human FECH was used at a dilution of 1 : 1000 and non-immune rabbit IgG was used as a negative control.

### Immunofluorescence analysis

A total of 5 × 10^5^ cells transfected with siRNA for 72 h were seeded onto a 10-well slide (2000 per well) and incubated at 37°C with 5% CO_2_. After 4 h, they were fixed with 4% paraformaldehyde for 20 min and 0.2% Triton X-100 for 5 min at room temperature. Immunostaining was performed by incubating the slides with mouse polyclonal anti-FECH antibody (1 : 500) at 4°C for 2 h, followed by incubation with Alexa Fluor 488 goat anti-mouse antibody (1 : 500; Invitrogen) for 1 h at room temperature in the dark. Finally, the sections were washed with Tris-buffered saline Tween-20 and cell images were captured on a fluorescence microscopy (AX 80, Olympus, Tokyo, Japan).

### Quantitative real-time–PCR (QRT–PCR)

Gene expression was quantified by QRT–PCR on a LightCycler using MasterPLUS SYBR Green (Roche Diagnostics, Indianapolis, IN, USA) as described previously (Nakada *et al*, 2006). The sense primer of human FECH (Genbank accession number NM_000140) was 5′-TGCACTCAAAATTTGCTTCG-3′ the antisense primer was 5′-GGCAAAACCCCATCTCTACA-3′ (amplicon size, 250 bp). Another primer set of FECH was also used: sense, 5′-GATGAATTGTCCCCCAACAC-3′ antisense, 5′-GCTTCCGTCCCACTTGATTA-3′ (amplicon size, 208 bp); *β*-actin (NM_001101): sense, 5′-CTACAATGAGCTGCGTGTGGC-3′ antisense, 5′-CAGGTCCAGACGCAGGATGGC-3′ (amplicon size, 271 bp). The PCR data were analysed by LightCycler analysis software as described previously (Nakada *et al*, 2006).

### Silencing of FECH with siRNA

The target sequence 1 of siRNA specific to human FECH was (2044–2064 bp) 5′-CCCAAGGGTAATAAACGTGTA-3′ sequence 2 was (1008–1028 bp) 5′-CCGACTGGTGTGGCAATCCAA-3′. The cells were transfected with either FECH siRNA or control luciferase using Lipofectamine 2000 (Invitrogen). The confluent cells seeded into 6-well plates were transfected with the respective siRNA and collected after 72 h by a scraper or with trypsin–EDTA. The effects of siRNA lasted for at least 96 h as determined by QRT–PCR and immunofluorescence analysis (Nakada *et al*, 2005).

### Western blot analysis

Western blot analysis was performed on the PVDF membrane according to the standard procedure as described previously (Nakada *et al*, 2005; Yoshida *et al*, 2009). Rabbit polyclonal antibody against FECH was used at a dilution of 1 : 1000.

### Protoporphyrin IX fluorescence molecular imaging

After transfection with FECH siRNA, the cells were harvested, counted, and plated (5 × 10^5^) on microscope coverslips in 100-mm dishes, and then incubated with serum-free DMEM with 12-h adherence. After adherence was achieved, the medium was immediately replaced with serum-free DMEM containing 5-ALA solution (final concentration: 400 *μ*M) (Hanania and Malik, 1992; Inoue *et al*, 2007; Miyake *et al*, 2009). After incubation for 6 h, cells on the coverslip were fixed with 4% paraformaldehyde in phosphate buffer solution (PBS) for 10 min at room temperature, washed with PBS twice, and mounted with mounting solution with another coverslip (Sinha *et al*, 2006). Protoporphyrin IX-specific fluorescence in the glioma cells was captured by fluorescence microscopy (BX 50, Olympus) and a digital camera (DP70, Olympus) under the same exposure time of the illumination source: phase-contrast images, 1/60 s, and PpIX molecular images, 20 s. Intracellular PpIX fluorescence was excited at 488 nm and molecular images were collected in the red channel through a 590-nm long-pass filter.

### Protoporphyrin IX fluorescence spectral intensity analysis

The transfected cells were harvested and counted, and 5 × 10^5^ cells were incubated with serum-free DMEM with 12-h adherence. After adherence was achieved, the medium was immediately replaced with serum-free DMEM containing 5-ALA solution (final concentration: 400 *μ*M) for 6 h at 37°C (Hanania and Malik, 1992). At the end of the incubation period, the cells were detached from the plates and centrifuged at 6 **g** and the pellets were resuspended in 100 *μ*l extraction solution (50 mM Tris-HCl, pH 7.5; 100 mM NaCl; 2.5% Triton X-100), shaken for 1 min at room temperature, and centrifuged at 1400 **g** for 5 min. The supernatant was transferred into 300 *μ*l ethyl acetate and glacial acetic-acid solution (v/v, 2 : 1). After 1-min shaking, the supernatant was added to 300 *μ*l of 0.6 M NaOH and centrifuged at 1400 **g** for 2 min, and the upper phase was dissolved in 300 *μ*l of 0.5 M HCl and centrifuged at 1400 **g** for 2 min. After centrifugation, the lower phase was collected and read for PpIX fluorescence using an SVLD-M1 (SBI ALApromo Co., Ltd, Tokyo, Japan; excitation, 400 nm; emission, 605 nm). Accessory software (BW-Spec V 3.26; Newark, NJ, USA) was used to analyse the PpIX fluorescence spectral intensity data. The two characteristic PpIX fluorescence spectras were detected, a sharp peak at 600–640 nm and a slightly broader peak at 700–710 nm as described previously (Loh *et al*, 1993). The PpIX fluorescence spectral intensity increased between 600 and 710 nm in a wide wave band due to exposure to the specific wavelength. We observed the two characteristic peaks (635 and 705 nm), indicating that the PpIX fluorescence was in the cells (Stummer *et al*, 1998a). Using the software, the steep rise in intensity at the 605-nm peak was used to calculate the difference in intensity at 600–640 nm of the cell line sample. The results are expressed as the mean of triplicate representative experiments (Utsuki *et al*, 2008).

### Cell proliferation assay

The Alamar Blue assay (Biosource, Camarillo, CA, USA) was used to assess cell proliferation as described previously (Nakada *et al*, 2005). After transfection with either control or FECH siRNA together treated with 400 *μ*M 5-ALA for 6 h, 5 × 10^5^ cells were exposed to two different fluences of light at 630 nm, 0.5 J cm^−2^ and 1 J cm^−2^, as described previously (Inoue *et al*, 2007; Madsen *et al*, 2009). After illumination, the medium was immediately replaced with DMEM containing 10% FBS. After incubation for 24 h at 37°C, 1000 cells of each population were seeded in 96-well plastic plates in 200 *μ*l of culture medium supplemented with 0.1% FBS (Hanania and Malik, 1992). The plates were incubated for 4 h at 37°C and Alamar Blue (Biosource) was added in a volume of 20 *μ*l (10% of total volume) to the cells and incubated. The plate was read on a fluorescence plate reader (excitation, 530 nm; emission, 590 nm) at 24, 48, 72, and 96 h. Average absorbance values were calculated.

### Cell apoptosis analysis

For analysis of apoptosis by 5-ALA–PDT, cells underwent the same pretreatment as described above in the cell proliferation assay. After incubation with DMEM containing 10% FBS for 24 h at 37°C, all cells were harvested and determined by staining with a human Annexin V FITC Apoptosis Detection Kit (Bender MedSystems, Burlingame, CA, USA), according to the manufacturer's instructions. Briefly, G112 and SNB19 cells were trypsinised, pelleted by centrifugation, and resuspended in Annexin V binding buffer. Fluorescein Isothiocyanate-conjugated Annexin V (1 *μ*g ml^−1^) and propidium iodide (20 *μ*g ml^−1^) were added to cells, which were incubated for 10 min at room temperature in the dark. At least 20 000 stained cells were analyzed on a FACS Caliber system (Becton Dickinson, Bedford, MA, USA) for each determination. The data were analyzed using Cell Quest software (Becton Dickinson).

### Statistics

Differences of FECH expression between various human astrocytic tumours and normal brain tissues were presented as mean±s.e. and analyzed by Welch's test using Graph Prism software (GraphPad Software, San Diego, CA, USA). Other data were expressed as mean (s.d.) and compared by Student's *t*-test. Probability values <0.05 were considered significant.

## Results

### Ferrochelatase mRNA expression and immunolocalisation in various human astrocytic tumours

**Figure 1:**
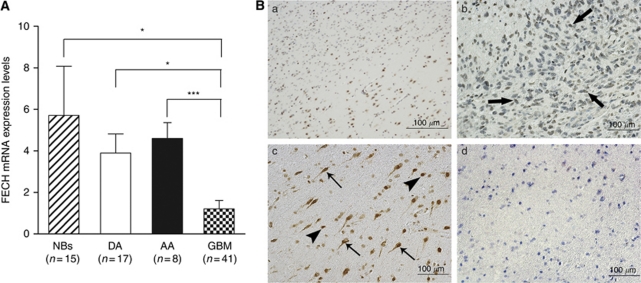
Expression levels of FECH mRNA in various human gliomas and immunolocalization of FECH in glioblastoma and normal brain tissues. (**A**) Relative mRNA expression levels of the *FECH* gene (FECH mRNA: *β*-actin mRNA ratios) in normal brains (NBs), diffuse astrocytomas (DA), anaplastic astrocytomas (AA), and glioblastomas (GBM) were analyzed by QRT–PCR. FECH mRNA expression levels were significantly lower in glioblastomas compared with normal brains, diffuse astrocytomas and anaplastic astrocytomas (^*^P<0.05; ^***^P<0.005). Data are expressed as means±s.e. (**B**) Immunolocalization of FECH in invading edge of glioblastoma (upper left: glioblastoma; lower right: normal brain, panel a), glioblastoma (panel b) and normal brain tissues (panels c and d). Paraffin sections were immunostained with anti-FECH antibodies (panels a, b and c) or non-immune rabbit IgG (panel d). Note that FECH is immunolocalized in the normal astrocytes (panel c, arrow head) and neurons (panel c, thin arrows). Faint staining is detected in neoplastic astrocytes (panel b, thick arrows), whereas no staining is observed in the normal brain with non-immune IgG (panel d).

The expression levels of FECH in human normal brain tissues (*n*=15) and glioma specimens (*n*=66) were evaluated by QRT–PCR using *β*-actin mRNA as an internal reference for normalisation (Figure 1A). Ferrochelatase expression seemed to be reduced in glioma tissues compared with normal brain tissues. The expression levels of FECH mRNA were significantly lower in glioblastoma tissues (mean±s.e., 1.21±0.40; *n*=41) than that in normal brain tissues (5.71±2.36; *P*<0.05; *n*=15), diffuse astrocytoma tissues (3.89±0.92; *P*<0.05; *n*=17) and anaplastic astrocytoma tissues (4.60±0.75; *P*<0.005; *n*=8).

Cells expressing FECH in normal brain and glioblastoma specimens were identified using immunohistochemistry. We can easily gain objective demarcation line between normal brain tissue and tumour tissue according to the density of FECH staining (Figure 1B, Panel a). Ferrochelatase was predominantly immunolocalised in the majority of neurons and astrocytes in normal brain specimens, whereas faint staining was detected in neoplastic astrocytes in glioblastoma specimens (Figure 1B). The density of FECH-positive cells was significantly lower in glioblastoma specimens compared with normal brain specimens under random fields of microscopic observation at the same magnification (Figure 1B, Panels b and c). This result was consistent with that of the QRT–PCR analysis.

### Ferrochelatase mRNA and protein expression in glioma cell lines

**Figure 2:**
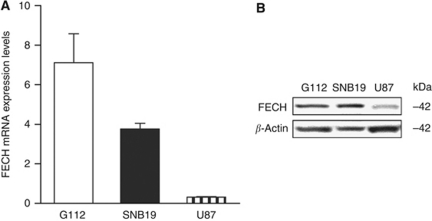
FECH mRNA and protein expression in glioma cell lines. (**A**) QRT–PCR was used to determine FECH mRNA expression levels in G112, SNB19, and U87 glioma cell lines. Data are expressed as mean (s.d.) of triplicate experiments. (**B**) Western blotting shows FECH protein in the glioma cell lines. The total cell lysates were immunoblotted with FECH and *β*-actin antisera.

To assess the efficiency of FECH siRNA in glioma cell lines, appropriate cell lines that express high levels of FECH should be chosen. Ferrochelatase mRNA expression in G112, SNB19, and U87 glioma cell lines was determined by QRT–PCR. The expression levels of FECH mRNA in these three glioma cell lines were significantly different, being highest in G112 and lowest in U87 (Figure 2A). Immunoblotting with a specific antibody against FECH was performed using the same cell lines. An ∼42-kDa single band of FECH (Ohgari *et al*, 2005) was clearly detected in the G112 and SNB19 cell lines, but faintly detected in U87 glioma cells (Figure 2B). This result was consistent with that of the QRT–PCR analysis. Thus, the G112 and SNB19 cell lines, in which FECH is highly expressed, were chosen for the FECH silencing experiment.

### Protoporphyrin IX fluorescence in glioma cell lines

**Figure 3:**
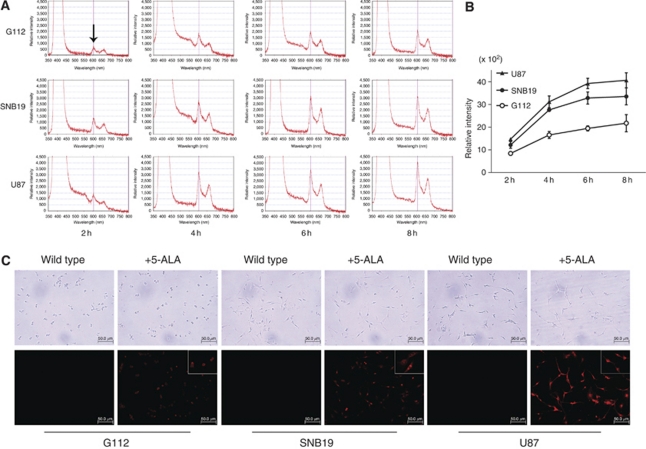
PpIX accumulation in glioma cells after 5-ALA exposure. (**A**) PpIX fluorescence spectra tracing at various time points. The peak at 605 nm (arrow) indicates the characteristic PpIX fluorescence spectra. *x*-axis: wavelength; *y*-axis: relative intensity. (**B**) The time-dependent PpIX accumulation curve was drawn according to the results of (**A**). PpIX fluorescence spectral intensity reached a plateau at ∼6 h in U87 and SNB19. The data are based on three independent experiments, and the error bars show mean (s.d.). (**C**) Molecular imaging of PpIX was visualised by fluorescence microscopy in G112, SNB19, and U87 cells that were incubated for 6 h with 5-ALA. Upper panels: phase-contrast images; lower panels: PpIX molecular images, insets: higher magnification.

To discern the optimal time point for exposing 5-ALA in glioma cell lines, time-dependent accumulation of PpIX was quantified. We detected a sharp peak at 605 nm, indicating the PpIX characteristic fluorescence spectra, in G112, SNB19 and U87 cells (Figure 3A). The spectral intensity of PpIX fluorescence increased in a time-dependent manner after 5-ALA exposure. In U87 and SNB19 cells, PpIX fluorescence intensity reached a plateau at ∼6 h (Figure 3B). Therefore, 6 h was chosen as the optimal time point for 5-ALA exposure. The intensity of PpIX fluorescence in U87 cells was higher than that in G112 and SNB19 cells, suggesting that U87 cells are more susceptible to 5-ALA than G112 and SNB19 cells. This is consistent with the data in Figure 2, showing a lower FECH expression in U87 cells than in G112 and SNB19 cells. Next, molecular imaging of PpIX was captured by fluorescence microscopy in the G112, SNB19 and U87 cells after 6-h 5-ALA exposure. PpIX fluorescence was not visualised in wild-type glioma cells without 5-ALA; however, PpIX-derived metabolic fluorescence was observed in the cytoplasm of the glioma cells after exposure to 5-ALA (Figure 3C). The degree of fluorescence intensity in the cell lines seemed to be consistent with the data in Figures 3A and B. These results indicate that exogenous exposure to 5-ALA increased accumulation of PpIX in glioma cells, which improved intensity of the fluorescence and that the intensity seems to be dependent of the endogenous level of FECH.

### Protoporphyrin IX accumulation after FECH silencing

**Figure 4:**
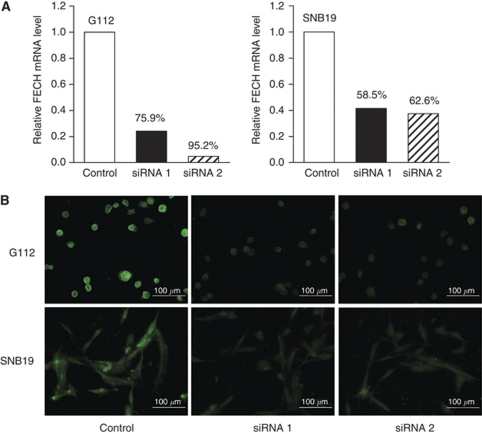
Efficiency of FECH siRNA silencing in glioma cell lines. (**A**) FECH siRNA sequence 1 and sequence 2 were transfected into G112 and SNB19 cells. Luciferase was transfected as a negative control, and total RNA was extracted from the cells 72 h later for cDNA synthesis. FECH mRNA expression after transfection was determined by QRT–PCR. (**B**) Micrographs of FECH protein labeled by green-fluorescent Alexa Fluor 488 dye (Invitrogen, Carlsbad, CA, USA) in G112 and SNB19 glioma cells transfected with siRNA. Green fluorescence intensity significantly decreased in FECH siRNA sequence 1 and sequence 2 compared with control siRNA. Representative micrographs of three independent experiments are shown.

To confirm the effect of FECH silencing, two siRNAs were transfected into SNB19 and G112 cells to specifically knockdown FECH mRNA expression. The mRNA levels of FECH at 72 h after siRNA transfection were determined by QRT–PCR. The knockdown ratio of FECH by both siRNA sequence 1 and sequence 2 was more than 50% in both cell lines (Figure 4A). Figure 4B shows FECH protein labelled by Alexa Fluor green fluorescence in G112 and SNB19 cells transfected with FECH siRNA or luciferase control. The decrease in green fluorescence intensity suggests depletion of FECH protein in cells transfected with FECH siRNA. These data suggest that sufficient FECH silencing was obtained using FECH siRNA sequence 1 and sequence 2.

**Figure 5:**
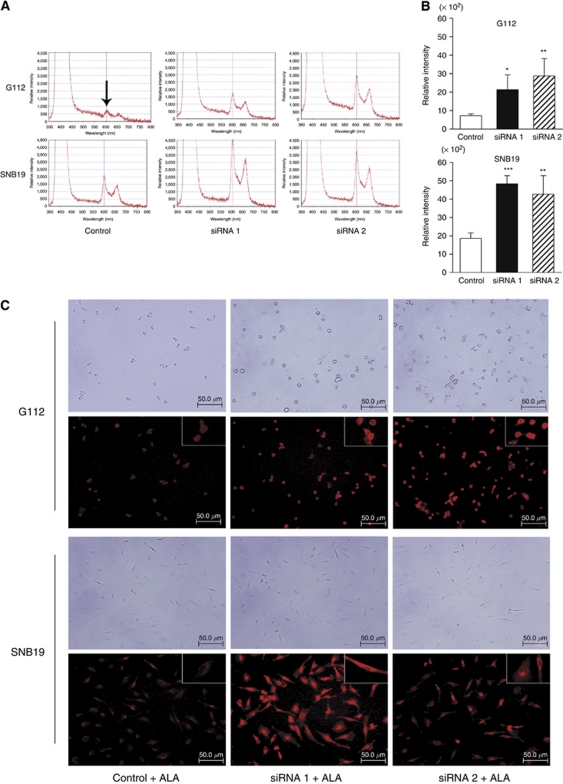
PpIX fluorescence spectra analysis and molecular images of glioma cells transfected with siRNA. (**A**) PpIX fluorescence spectra tracing at 6 h after FECH or luciferase siRNA transfection and incubation with 400 *μ*M 5-ALA. The peak at 605 nm (arrow) indicates the characteristic PpIX fluorescence spectra. *x*-axis: wavelength; *y*-axis: relative intensity. (**B**) Histogram of PpIX relative fluorescence intensity of cells transfected with siRNA after 6-h exposure to 400 *μ*M 5-ALA. PpIX fluorescence spectral intensity was significantly increased in the cells transfected with FECH siRNA sequence 1 and sequence 2 compared with that in the cells transfected with control siRNA (^*^P<0.05; ^**^P<0.01; ^***^P<0.005). Data are expressed as mean (s.d.) of triplicate experiments. (**C**) PpIX molecular images of glioma cells transfected with FECH siRNA. Molecular images of PpIX were visualized by fluorescence microscopy in G112 and SNB19 glioma cells. After 72-h FECH siRNA transfection, the cells were exposed to 400 *μ*M 5-ALA for 6 h. Note that strong red fluorescence of glioma cells was observed in cells transfected with FECH siRNA compared with control siRNA. Upper panels: phase-contrast images; lower panels: PpIX molecular images, insets: higher magnification.

Next, to investigate whether FECH interference causes abundant accumulation of PpIX after 5-ALA exposure, cells transfected with control or FECH siRNA were treated with 5-ALA. Expectedly, PpIX fluorescence spectral intensity was significantly increased in cells transfected with FECH siRNA sequence 1 and sequence 2 compared with control luciferase-siRNA (Figures 5A and B). The increasing ratio of PpIX fluorescence spectral intensity after FECH interference is higher in G112, which was more sensitive to FECH siRNA than SNB19. In G112 cells, transfection of siRNA sequence 2 (mean±s.d., 28.80±4.70) induced a higher level of PpIX fluorescence spectral intensity, ∼400% of the control (7.27±0.48; *P*<0.01). In SNB19 cells, the effect of siRNA sequence 1 (48.36±2.23) and sequence 2 (42.65±5.10) was similar in that the relative intensity of transfection with FECH siRNA was 200% greater than that of the control (18.61±1.52, *P*<0.005; *P*<0.01, respectively, Figure 5B). These data demonstrated that depletion of FECH resulted in a significant increase of intracellular PpIX accumulation in glioma cells after 5-ALA exposure.

### Ferrochelatase silencing enhances PpIX molecular imaging quality

We then investigated whether FECH silencing enhances PpIX molecular imaging quality. We captured fluorescence images of cells transfected with siRNA using fluorescence microscopy after exposure to 5-ALA for 6 h. Bright and clear red fluorescence were visualised in glioma cells transfected with FECH siRNA compared with control (Figure 5C). High-contrast strong red fluorescence was diffusely localised to the cytoplasm of glioma cells transfected with FECH siRNA. Furthermore, we can clearly discriminate between glioma cell cytoplasm and nuclei at higher magnification. These data indicate that we can obtain high molecular imaging quality of PpIX fluorescence in glioma cells by silencing FECH.

### Ferrochelatase knockdown enhances 5-ALA-based PDT efficacy

**Figure 6:**
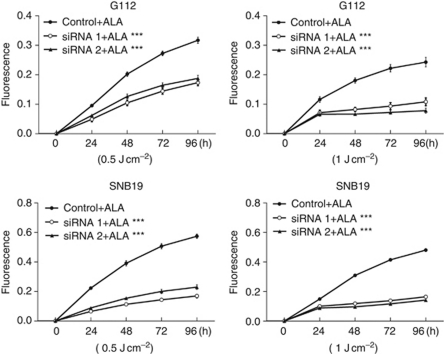
Proliferation curve of glioma cells transfected with FECH siRNA after light irradiation. Luciferase and FECH siRNA sequence 1 and sequence 2 were transfected into G112 and SNB19 cells for 72 h and then cells were exposed to 400 *μ*M 5-ALA for 6 h. The cells were irradiated with 0.5 J cm^−2^ or 1 J cm^−2^ light fluence and then analyzed using Alamar Blue assay (Biosource, Camarillo, CA, USA). Data are expressed as mean (s.d.) of octuple experiments (^***^P<0.005).

Furthermore, we performed a proliferation assay to examine the influence of FECH silencing on 5-ALA–PDT. According to the proliferation curve diagram, light-irradiated cells transfected with FECH siRNA showed a pronounced inhibitory effect on cell proliferation in both G112 and SNB19 cells (*P*<0.005, Figure 6). Furthermore, proliferation was suppressed more with 1 J cm^−2^ light fluence than with 0.5 J cm^−2^ light fluence in both cell lines. These data suggest that FECH knockdown enhances the PDT effect in a light fluence-dependent manner.

**Figure 7:**
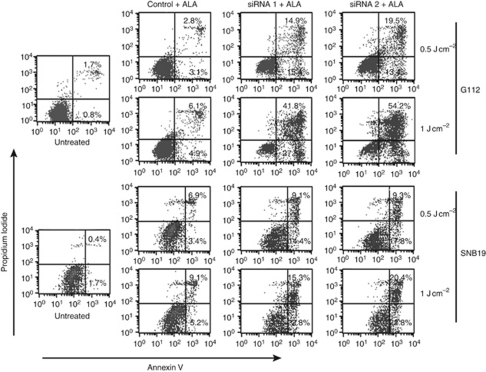
Apoptosis analysis of glioma cells transfected with FECH siRNA after light irradiation. Cells were harvested 24 h after transfection and light irradiation and then subjected to combined staining with propidium iodide and Annexin V. The number in each quadrant indicates the proportion of the cells that are present in the quadrant. Results are representative of three independent experiments.

Apoptosis was quantified by flow-cytometric analysis. The results show that FECH silencing significantly increased the proportions of both early (Annexin V-positive, PI-negative) and late (Annexin V-positive, PI-positive) apoptotic cells induced by 5-ALA–PDT. In G112 cells with 0.5 J cm^−2^ light fluence, the proportion of early apoptotic cells was 15.4 and 13.4% in the FECH siRNA sequence-1 and sequence-2 group, respectively, much more than the 3.1% seen in the negative control (Figure 7). Transfected SNB19 cells responded similarly. Furthermore, the number of apoptotic cells was significantly higher with 1 J cm^−2^ light fluence than with 0.5 J cm^−2^ light fluence. These results suggest that 5-ALA–PDT enhancement by FECH knockdown can be attributed to induction of apoptosis.

## Discussion

This study has demonstrated significant downregulation of FECH mRNA expression in glioblastomas compared with normal brain tissues. Studies have shown that low expression or molecular defects of FECH in malignant tumours, such as colon cancer and urothelial cancer, is correlated with intracellular PpIX accumulation (Peng *et al*, 1997; Miyake *et al*, 2009). Currently, no data have been reported concerning the metabolism of 5-ALA in glioma, although 5-ALA fluorescence-guided glioma resection is well established. In this study, we showed that SNB19 cells, which expressed lower levels of FECH than G112 cells, accumulated more PpIX than G112 cells after exposure to 5-ALA. Ferrochelatase silencing in SNB19 and G112 cells promoted PpIX accumulation and enhanced visible red fluorescence *in vitro*. Together, the *in vitro* and *in vivo* data indicate a strong correlation between FECH expression and PpIX accumulation. Our results are consistent with clinical observations of glioblastomas commonly showing high-intensity PpIX fluorescence. Thus, this study is the first to reveal that FECH has a role in the metabolism of 5-ALA in glioma.

It remains possible that other molecules, not studied here, may contribute to the metabolism of 5-ALA in glioma. Pre-clinical and clinical studies suggest that the accumulation of PpIX in glioblastoma cells may be caused by various factors (Norum *et al*, 2009). As the normal blood–brain barrier (BBB) is impermeable to 5-ALA, the compromised BBB in glioblastoma tissue is necessary for 5-ALA to cross BBB to get to the glioblastoma cells. 5-Aminolevulinic acid has to cross the tumour cells through the transporters, such as peptide transporter 2 (PEPT2), which is the primary transporter responsible for the astrocytic uptake of 5-ALA (Rodriguez *et al*, 2006; Xiang *et al*, 2006). Actually PEPT2 is overexpressed in glioblastoma cells (our unpublished data). Several molecules are known to be involved in the heme synthesis pathway in tumour cells (Ennis *et al*, 2003). A previous report has indicated that decreased PpIX levels and therapeutic effect of 5-ALA–PDT were induced by the silencing of heme oxygenase (HO) (Miyake *et al*, 2009). Analysis of responsible molecules for the metabolism of 5-ALA in glioma cells is ongoing in our laboratory.

Objective quantification of PpIX is critical to assess the changes in PpIX fluorescence intensity, but it is difficult to achieve. In this study, we measured intracellular PpIX using fluorescence spectral intensity analysis, which allows an observer to check the specified PpIX spectra on a monitor by reading the PpIX fluorescence wavelength peak. Compared with spectrofluorometric (Sinha *et al*, 2006; Miyake *et al*, 2009) and flow-cytometric analysis (Ickowicz Schwartz *et al*, 2004; Inoue *et al*, 2007), this analysis has prominent advantages for PpIX fluorescence quantification: (1) directly and objectively, it has a characteristic spectra tracing that indicates specified fluorescence (e.g., the sharp peak at 605 nm indicative of the characteristic PpIX fluorescence spectra); (2) it is simple procedure and requires only that an investigator recognise the spectra and read the result; and (3) it is a potential diagnostic tool to assist with fluorescence-guided resection. Ours and recent other studies suggest that the usefulness of fluorescence spectral intensity analysis (Utsuki *et al*, 2006, 2008) may facilitate PpIX recognition by this system in both pre-clinical and clinical settings.

With our convincing evidence of a causal relationship between FECH expression and PpIX accumulation, we assumed that the FECH enzyme may function as a promising molecular target to increase PpIX accumulation for clinical application. As we expected, FECH siRNA caused abundant PpIX accumulation and more visible red fluorescence in glioma cells, suggesting that FECH silencing can improve PpIX fluorescence discrimination accuracy. Depending on the high-contrast intensity and high-resolution imaging, neurosurgeons can potentially obtain the best detailed diagnostic information for facilitating fluorescence recognition and localisation.

Furthermore, we assessed the effect of FECH silencing on 5-ALA-PDT efficacy. Generally, PDT efficacy depends on parameters, such as photosensitizing agent, light fluence, illumination time, oxygen, photosensitizer concentration, and intrinsic tissue sensitivity to the PDT effect (Sinha *et al*, 2006; Madsen *et al*, 2009). In our study, FECH siRNA significantly enhanced 5-ALA-PDT efficacy in glioma cells caused by marked growth inhibition and abundance of apoptotic cells under unchanged above parameters except for photosensitizer (PpIX) concentration. It is highly likely that intrinsic PpIX level is critical for the 5-ALA-PDT effect and that the radical cause of enhanced 5-ALA–PDT by FECH silencing is the high levels of PpIX. Currently, the exact mechanism of 5-ALA–PDT is not known. In our study, accumulated PpIX within the glioma cells may have caused irreversible photodamage by exposure to a specific wavelength. Recent studies have shown that 5-ALA–PDT-induced photodamage causes mitochondrial and nuclear DNA damage; subsequently, massive apoptosis occurs because of mitochondrial release of cytochrome *c* and activation of caspase in glioma cells (Inoue *et al*, 2007; Karmakar *et al*, 2007). Further studies will be required to explore the detailed mechanism of 5-ALA–PDT-induced photodamage in human glioma cells.

RNA interference (RNAi) offers great potential, not only for *in vitro* target validation but also *in vivo* as a novel therapeutic strategy based on highly specific and efficient silencing of a target gene in tumour therapy (Grzelinski *et al*, 2006). This study is the first to demonstrate that FECH siRNA, which is a non-invasive and safe molecular agent because it led to an accumulation of intrinsic and non-toxic PpIX within the cytoplasm, offers the promise of being an exciting new tool with great potential to enhance the efficiency of 5-ALA fluorescence and 5-ALA–PDT in glioma cells. However, at the present time, application of siRNAs *in vivo* is severely limited by their instability and poor delivery into target cells and target tissues (Guo *et al*, 2010). Currently, various potential methods for delivering siRNA into target cells have been developed. Pardridge and his collaborators have applied immunoliposomes containing a short hairpin RNA (shRNA) expression vector against the epidermal growth factor receptor (EGFR) to suppress brain tumour proliferation with a single intravenous injection in adult rats with intracranial brain tumours (Pardridge, 2004). In that system, two monoclonal antibody ligands on liposomes were used for delivering shRNA into specific tumour cells, one against the transferrin receptor that exists on the BBB, and the other against the insulin receptor that was used for subsequent targeting of the liposomes to the brain tumour cells (Mathupala *et al*, 2006). Further studies are required for development of RNAi-specific delivery systems and exploration of high affinity receptors for glioma cells but not normal brain tissue before clinical application.

In conclusion, we have demonstrated for the first time that FECH siRNA enhanced the quality of 5-ALA fluorescence imaging for optimising subjective fluorescence discrimination and improved the efficacy of 5-ALA–PDT by increasing intrinsic PpIX levels. The best way to enhance 5-ALA fluorescence quality and 5-ALA–PDT efficacy in a real-time intraoperative manner *in vivo* is currently under investigation in our laboratory.
